# Enthesopathy, Osteoarthritis, and Mobility in X-linked Hypophosphatemia^1^

**DOI:** 10.1210/clinem/dgaa242

**Published:** 2020-05-06

**Authors:** Erik A Imel

**Affiliations:** Department of Medicine and Pediatrics, Division of Endocrinology, Indiana University School of Medicine, Riley Hospital for Children, Indiana Center for Musculoskeletal Health, Indianapolis, Indiana

X-linked hypophosphatemia (XLH) is a rare bone disease affecting an estimated 1:20 000 to 25 000 individuals. XLH is caused by mutations in *PHEX* whose key consequences include elevations of fibroblast growth factor 23 (FGF23) causing renal phosphate losses and impairments of vitamin D activation with resultant hypophosphatemic rickets and osteomalacia (1). Additional molecular consequences of PHEX deficiency (beyond FGF23, hypophosphatemia, and vitamin D metabolism) may contribute to disease features including dental abscesses. Conventional medical therapy with active forms of vitamin D plus phosphate salts improve skeletal deformities in growing children and osteomalacia symptoms in adults. Recently anti-FGF23 antibody treatment using burosumab has become available.

Although usually diagnosed in childhood, despite conventional therapy the lifelong consequences of XLH often include short stature, skeletal deformities, significant pain, impaired mobility, and disability among adults, which impair quality of life (1, 2). Impaired mobility begins in childhood, but in adults is largely a mechanical consequence of residual lower extremity deformities, enthesopathy, and osteoarthritis, along with bone pain from osteomalacia and pseudofractures. About 50% of adults with XLH had active fractures or pseudofractures (3). Enthesopathy affects nearly 100% of adults with XLH eventually, increasing with age (3-5). Enthesopathy begins with the calcification of tendon and ligament insertion sites but progresses to development of osteophytes, often bridging between adjacent bones (4-6). Enthesopathy is usually bilateral and predominantly affects weight bearing joints and the spine, where it can cause spinal stenosis. Enthesopathy is not known to be prevented by, or responsive to, any medical therapy. Osteoarthritis is highly prevalent in adults with XLH, affecting 63% (3) and beginning at younger ages than in the general population. Osteoarthritis is most likely a consequence of lifelong abnormal mechanical loading of joints resulting from the skeletal deformities remaining from childhood.

In this issue, Steele et al. performed detailed skeletal and functional assessments in a cross-sectional study of 9 adults younger than age 60 years with XLH (6). Ambulatory subjects were selected having self-reported functional disability. Thus, subjects without reported disability, and hence more mild disease, were excluded. On the other hand, subjects with extreme disability causing inability to walk at least 200 feet were also excluded. This study was small and did not encompass the full the range of mobility and function of adults with XLH, and hence could not estimate the prevalence of dysfunction. However, the detailed measurements provide important insights into the features influencing mobility among adults with XLH.

All participants reported bone and joint pain. Radiographic evidence of osteoarthritis and enthesopathy were extensive, affecting all major joints of the upper and lower extremities. Enthesopathy throughout the spine included the anterior and posterior spinal ligaments. Most had kyphosis and scoliosis. Osteoarthritis and enthesopathy were especially prominent around the pelvic girdle, along with flattening of the femoral head, coxa vara, and bowing of the femur shaft. These features are commonly reported in XLH, though the extent of radiographic involvement in these adults is notable when considering the disability associated with this disease.

Balance scores and strength on manual motor testing were normal, but scores on a patient reported lower extremity functional scale were significantly lower than controls. Passive range of motion was limited at the hip, knee, and ankle. Cervical spine extension was impaired, whereas flexion was spared. These findings have consequences for mobility and gait.

This was the first study to report kinematic gait analysis to characterize gait abnormalities in XLH using reflective markers over bony landmarks and video-recording subjects as they walked. Joint angles during gait were smaller in XLH subjects consistent with the passive range of motion testing. The rigidity of the spine resulting from enthesopathy corresponded to a more flexed position throughout the gait cycle and a fixed stooped posture. XLH subjects had greater bilateral sway of the trunk, whereas limitations in hip and knee extension resulted in shorter steps. Altogether, these individual abnormalities quantitatively characterize the classic waddling gait of XLH.

This study moved from the radiographic description of osteoarthritis and enthesopathy in adults with XLH to quantitate their impact on range of motion and gait, which, along with pain, are critical to function for activities of daily living. Abnormal joint motions during gait may further contribute to pain, arthritis, and dysmobility in an ongoing cycle. Further, the upper extremity findings would result in additional compromise of activities of daily living beyond those involving sitting, standing, and walking.

Further studies are needed to establish the timing and rate of progression of enthesopathy. Connor et al. quantified enthesopathy by listing number of affected sites, and found there was no apparent effect of proportion of time treated with conventional therapy (4). However, this method does not account for differences in the amount of enthesopathy at an individual location. A reliable method is needed to quantify enthesopathy and osteophytes both in the individual bony locations and in the total patient.

Mouse models suggested that FGF23 itself might be directly involved in enthesopathy and that mineralization of entheses might be exacerbated by treating with calcitriol and phosphate (5, 7). Although this suggests that strategies to block the effects of FGF23 hypothetically could be beneficial, there are no studies in mice or humans addressing this question. Specifically, there currently are no data to determine whether anti-FGF23 antibody therapy (such as with burosumab) might have any effect to slow the progression of enthesopathy. In a placebo controlled trial in 134 adults with XLH, burosumab improved self-reported ratings of stiffness and physical function, but no difference between groups was seen for the 6-minute walk distance after 24 weeks, possibly because of the pervasive osteoarthritis and enthesopathy and the relatively short time frame, though in the extension study with all subjects receiving burosumab, by week 48, subjects had increased 6-minute walk distance (3).

In a randomized controlled trial, 61 children with XLH and having persistent rickets despite conventional therapy had improvements in rickets severity and lower limb deformity when switched to treatment with burosumab compared with ongoing conventional therapy (8). Improvements of lower limb deformity might decrease risk for osteoarthritis. However, the full magnitude of impact of burosumab during the growing years is not known. Pediatric trials of burosumab were limited to children between the ages of 1 and 12 at enrollment and mostly enrolled prepubertal children. Clinical trials have not assessed the effects of burosumab during puberty or during the transition to adulthood. Few children have been treated through pubertal completion and none for the full duration from infancy to the end of the growth period. Consequently, the magnitude of potential effect and advantage over conventional therapy on lower limb deformity and hence the mechanical effect on joints as patients enter adulthood is not yet known.

Currently, adequate evidence is lacking to support the best physical treatment approaches (eg, physical or occupational therapy, surgical approaches, etc.) to enthesopathy and osteoarthritis in adults with XLH. In addition, the effects of novel medications for XLH on enthesopathy or osteoarthritis have not been assessed. It is important to be able quantitate the disease burden of adults with XLH and the underlying mechanical abnormalities involved so that the effects of medical or nonmedical interventions can be assessed. Enthesopathy and osteoarthritis develop gradually over the course of many years. Years of treatment, along with appropriate comparison groups, will be necessary to clearly identify whether new treatment options impact development of these features.

## Additional Information

***Disclosure Summary*:** The author has received research funding from Ultragenyx Pharmaceuticals as a clinical trial investigator and has served on advisory boards.

## References

[1] Haffner D, Emma F, Eastwood DM, et al. Clinical practice recommendations for the diagnosis and management of X-linked hypophosphataemia. Nat Rev Nephrol. 2019;15(7):435-455. 10.1038/s41581-019-0152-5 PMC7136170 31068690

[2] Skrinar A, Dvorak-Ewell M, Evins A, et al. The lifelong impact of X-linked hypophosphatemia: results from a burden of disease survey. J Endocr Soc. 2019;3(7):1321-1334. 31259293 10.1210/js.2018-00365 PMC6595532

[3] Portale AA, Carpenter TO, Brandi ML, et al. Continued beneficial effects of burosumab in adults with X-linked hypophosphatemia: results from a 24-week treatment continuation period after a 24-week double-blind placebo-controlled period. Calcified Tissue Int. 2019;105(3):271-284. 10.1007/s00223-019-00568-3 31165191

[4] Connor J, Olear EA, Insogna KL, et al. Conventional therapy in adults with X-linked hypophosphatemia: effects on enthesopathy and dental disease. J Clin Endocrinol Metab. 2015;100(10):3625-3632. 26176801 10.1210/JC.2015-2199 PMC4596038

[5] Liang G, Katz LD, Insogna KL, Carpenter TO, Macica CM Survey of the enthesopathy of X-linked hypophosphatemia and its characterization in Hyp mice. Calcified Tissue Int. 2009;85(3):235-246. 10.1007/s00223-009-9270-6 PMC2988401 19609735

[6] Steele A, Gonzalez R, Garbalosa JC, et al. Osteoarthritis, osteophytes, and enthesophytes affect biomechanical function in adults with X-linked hypophosphatemia. J Clin Endocrinol Metab. 2020;105(4):e1798-e1814. 10.1210/clinem/dgaa064 PMC8416779 32047911

[7] Karaplis AC, Bai X, Falet JP, Macica CM Mineralizing enthesopathy is a common feature of renal phosphate-wasting disorders attributed to FGF23 and is exacerbated by standard therapy in hyp mice. Endocrinology. 2012;153(12) :5906-5917. 23038738 10.1210/en.2012-1551 PMC3512070

[8] Imel EA, Glorieux FH, Whyte MP, et al. Burosumab versus conventional therapy in children with X-linked hypophosphataemia: a randomised, active-controlled, open-label, phase 3 trial. Lancet. 2019;393(10189):2416-2427. 31104833 10.1016/S0140-6736(19)30654-3 PMC7179969

